# New insights into Wiskott-Aldrich syndrome: ten novel *WAS* mutations and their clinical impact in a Brazilian cohort

**DOI:** 10.3389/fimmu.2025.1585594

**Published:** 2025-07-31

**Authors:** Lucas W. Santos, Samuel S. Medina, Jéssica O. Frade-Guanaes, Lúcia H. Siqueira, Luiz Gustavo R. de Lima, Bruna Chati, Marcos T. Nolasco da Silva, Adriana G. L. Riccetto, Paula Lyra, Ana Carla A. M. Falcão, Pedro P. A. Santos, Regina S. W. Di Gesu, Bianca Stefanello, Gabriela G. Yamaguti-Hayakawa, Carmem M. S. Bonfim, Maria M. S. Vilela, Margareth C. Ozelo

**Affiliations:** ^1^ Hemocentro UNICAMP, University of Campinas, Campinas, SP, Brazil; ^2^ Department of Internal Medicine, School of Medical Sciences, University of Campinas, UNICAMP, Campinas, SP, Brazil; ^3^ Center for Investigation in Pediatrics, School of Medical Sciences, University of Campinas, UNICAMP, Campinas, SP, Brazil; ^4^ Department of Pediatrics, School of Medical Sciences, University of Campinas, UNICAMP, Campinas, SP, Brazil; ^5^ Department of Pediatrics, Professor Fernando Figueira Institute of Integral Medicine (IMIP), Recife, PE, Brazil; ^6^ Department of Pediatrics, Conceição Children’s Hospital (HCC), Porto Alegre, Brazil; ^7^ Pediatric Blood and Marrow Transplantation Unit, Complexo Hospital de Clínicas, Universidade Federal do Paraná, Curitiba, Brazil

**Keywords:** Wiskott-Aldrich syndrome, WAS, WASp mutation, thrombocytopenia, inborn errors of immunity

## Abstract

**Background:**

Wiskott-Aldrich Syndrome (WAS) is a rare and severe X-linked immunodeficiency disorder characterized by microthrombocytopenia, eczema, and increased susceptibility to infections, autoimmunity, and malignancies. This study aims to explore molecular changes in the WAS gene in Brazilian patients and assess their correlation with clinical manifestations and disease severity.

**Methods:**

Thirty-one patients from 27 families with thrombocytopenia suspected to have WAS or X-linked thrombocytopenia (XLT) were analyzed. Clinical evaluation, cell morphology analysis, and flow cytometry (when feasible) were performed. DNA samples underwent direct sequencing to identify WAS gene mutations.

**Results:**

Genomic sequencing identified 17 WAS gene variants, 10 of which were novel, expanding the genetic diversity of the disorder. The most frequent *WAS* gene variants were primarily frameshift indels that introduced premature stop codons, with five localized in exon 10. While thrombocytopenia and small platelets were prevalent, atypical presentations, including one patient with normal platelet size, were observed. The correlation between genotype and phenotype was complex, as some patients harboring similar mutations demonstrated varying disease severities. Of the 22 confirmed cases, 12 underwent hematopoietic stem cell transplantation (HSCT), while six succumbed to severe disease complications, including opportunistic infections and malignancies.

**Conclusions:**

The study underscores the need for early molecular diagnosis and tailored treatments, particularly HSCT, which remains the standard curative therapy. Additionally, the findings emphasize the role of genetic variation in predicting disease severity, underlining the importance of personalized medical approaches for WAS patients.

## 1 Introduction

Wiskott-Aldrich Syndrome (WAS) is a rare, X-linked recessive disorder characterized by microthrombocytopenia, immunodeficiency, eczema, and an increased predisposition to autoimmunity and malignancies (1). The condition is caused by mutations in the Wiskott-Aldrich syndrome protein (WASp), which is critical in cytoskeletal regulation and immune cell function. WASp is predominantly expressed in hematopoietic cells, where it is essential for actin cytoskeleton remodeling, immune synapse formation, and effective immune responses (2–4). Defective WASp function impairs immune signaling, leading to immune dysregulation and a heightened risk of infections, bleeding disorders, and inflammatory complications (5, 6).

The clinical presentation typically emerges within the first few months of life, with affected infants presenting with petechiae, eczema, and recurrent skin infections (7). The progression of the disease can include recurrent bacterial infections, like otitis and pneumonia, and an increased risk of sepsis. Persistent immune dysregulation, along with elevated gamma globulin levels, further increases the risk of developing autoimmunity and malignancies (8).

Variations in the *WAS* gene exhibit significant diversity, including missense changes, splicing defects, deletions, and nonsense mutations (9, 10). The phenotypic spectrum of WASp variation extends beyond classic WAS to milder forms such as X-linked thrombocytopenia (XLT), with disease severity closely tied to the nature and location of the mutation (11). The clinical overlap between WAS and conditions like immune thrombocytopenia (ITP) can lead to misdiagnosis, making molecular diagnostics crucial for accurate identification and timely treatment (12). For instance, a study of 78 children initially diagnosed with ITP found that 43.6% were later diagnosed with WAS, underscoring the importance of genetic analysis, particularly for males with early onset and low mean platelet volume (MPV) (13).

Hematopoietic stem cell transplantation (HSCT) remains the gold standard for curative treatment of WAS, with the best outcomes typically observed when the procedure is performed before the age of five, yielding a survival rate of nearly 90% (14–16). More recently, autologous hematopoietic stem cell (HSC) gene therapy has emerged as a promising alternative, offering a potential cure without the need for a matched donor and avoiding the risks associated with traditional HSCT, such as graft-versus-host disease (GvHD) (17).

To facilitate diagnosis and clinical decision-making, a standardized scoring system was initially developed (10) and later refined (18, 19), providing a consistent approach to assess disease severity (**Supplementary Table S1**). Thrombocytopenia is a hallmark of WAS, and more severe cases often involve complications such as autoimmunity and malignancy. Recent studies, including by Albert et al. (2022) (16), have identified a subgroup of patients with early-onset disease (before age two) who present with life-threatening symptoms such as severe refractory thrombocytopenia, emphasizing the need for earlier intervention.

Recent outcome analyses have suggested that the *WAS* gene variation class, including the locus and type of variant, may serve as a predictive biomarker for disease severity and complications (20). This insight may guide the timely initiation of curative therapies such as HSCT or HSC gene therapy.

Given the clinical heterogeneity of WAS, integrating molecular diagnostics with clinical phenotyping is critical to improving patient outcomes. Identifying novel WASp mutations and their associated phenotypic presentations will enhance our understanding of genotype-phenotype correlations in WAS. This study aims to investigate molecular alterations in patients with suspected WAS and correlate these findings with clinical manifestations and disease severity scores.

## 2 Materials and methods

### 2.1 Patient selection and data collection

This study included individuals diagnosed with thrombocytopenia, either characterized by small platelets or associated with recurrent infections. All participants were referred to the Hematology Center (Hemocentro UNICAMP) at the University of Campinas for molecular analysis of the WAS gene. Additionally, patients referred by the Brazilian Group for Immunodeficiency (BRAGID) and the Latin American Society for Immunodeficiencies (LASID) were included. Only cases with confirmed genetic diagnosis were considered for inclusion in this study.

In addition to molecular diagnosis, comprehensive clinical data were collected to facilitate genotype-phenotype correlation. Patients were assigned a disease severity score based on clinical manifestations, following previously established criteria (**Supplementary Table S1**) (10, 19, 21). All participants and/or their caregivers provided written informed consent, and the study was conducted in accordance with ethical guidelines and institutional review board (IRB) approvals. The study received approval from the University of Campinas Ethics Committee (CAAE: 24548313.1.0000.5404).

### 2.2 Nucleic acid isolation and cDNA synthesis

Genomic DNA (gDNA) was extracted from whole blood by TKM buffer (Tris-HCL 10 mM pH 7.6; KCL 10 mM; MgCl_2–_10 mM; EDTA 20 mM) and SDS. Total RNA was extracted by Trizol (Life Technologies, Grand Island, NY) and then submitted to reverse transcriptase-polymerase chain reaction (RT-PCR) using the RevertAid H minus First Strand complementary DNA (cDNA) Synthesis Kit (Thermo Fisher Scientific, MA, EUA) according to manufacturer’s instructions.

### 2.3 Polymerase chain reaction amplification and sequence analysis

PCR amplification of gDNA or cDNA was performed using custom-designed primers targeting the *WAS* gene (RefSeq: NG_007877.1, NM_000377.3) and the WASP-interacting protein (WIP) gene (*WIPF1*, RefSeq: NG_032009.1, NM_001077269.1) as detailed in **Supplementary Table S2**. Direct sequencing was performed using the ABI PRISM^®^ 3500 Genetic Analyzer (Applied Biosystem, Thermo Fisher Scientific, EUA), and chromatograms were generated on Chromas^®^. All patients underwent sequencing of the *WAS* gene, and for those without detectable variations associated with WAS or XLT, sequencing of the *WIPF1* gene was also carried out. The nomenclature of the identified variants was structured according to HUGO/HGVS recommendations (22).

### 2.4 Flow-cytometric analysis of WASp expression

The procedure was performed according to previously standardization protocols (23). Intracellular WASp expression was evaluated in 200 μl peripheral whole blood or peripheral blood mononuclear cells (PBMC), using Fix&Perm^®^ Cell Permeabilization Kit (Becton Dickinson) according to the manufacturer’s recommendations. Cells were incubated with a mouse anti-WASP monoclonal antibody (Clone 7B10E4, Thermo Fisher Scientific, MA, USA), followed by a FITC-conjugated goat anti-mouse IgG secondary antibody (Thermo Fischer Scientific), diluted 1:1200. Incubation was carried at room temperature for 15 minutes. Data acquisition was performed using a FACSCalibur^™^ flow cytometer, and analysis was conducted with FlowJo^®^ software (Becton Dickinson).

### 2.5 Statistical analysis

Descriptive statistical analysis was conducted for nominal variables. Continuous variables were summarized using median and range (min-max). Categorical variables were expressed as percentages. The correlation of numerical variables was assessed using Spearman’s correlation coefficient, and a significance level of 5% was adopted. All statistical analyses were conducted using GraphPad Prism 10.4.1 (GraphPad Software, San Diego, CA, USA).

## 3 Results

In this study, 31 patients from 27 families were screened for molecular changes in *WAS* gene. Of those, 22 patients from 17 families received the molecular diagnostic confirmation through *WAS* gene DNA-sequencing, and three siblings were diagnosed post-mortem after confirmation via maternal genotyping (**Table 1**). The median age at diagnosis was 16 months (range: 2–271 months). All patients exhibited moderate to severe thrombocytopenia, with a median platelet count of 21.5 x 10^9^/L (range: 5 to 120 x 10^9^/L).

**Table 1:** Patient’s demographic and clinical characteristics.

Patients’ Characteristics	n = 22
Median age at diagnosis, months (min-max)	16 (2-271)
Platelet count (10^9^/L), median (min-max)	20 (5-120)
Mean platelet volume (MPV), median (min-max)	6.5 (4.3-12.9)
Symptoms, n (%)	
Eczema	15 (71.4)
Hematochezia	12 (57.1)
Petechiae/Bruise	12 (57.1)
Epistaxis	9 (42.9)
Recurrent Infections	7 (33.3)
Cutaneous-mucous bleeding	6 (28.6)
Skin infections	6 (28.6)
Respiratory Infection	5 (23.8)
Allergies	4 (19)
Outcome, n (%)	
Post-HSCT	12 (54.5)
Death	6 (27.3)
Waiting for HSCT	2 (9.1) ^*^
Follow-up	2 (9.1) ^**^
Clinical Score, n (%)	
1-2 (XLT)	2 (9.1)
3 (WAS)	8 (36.4)
4 (WAS)	6 (27.3)
5 (WAS)	6 (27.3)
Variation effect, n (%)	
*Frameshift*	14 (63.6)
*Missense*	5 (22.7)
*Nonsense*	2 (9.1)
*Splice site*	1 (4.5)

^*^Patients recently diagnosed are currently being prepared for HSCT. ^**^Patients diagnosed with XLT have, to date, chosen not to proceed with HSCT, following a shared decision-making process. MVP, mean platelet volume; WAS, Wiskott-Aldrich syndrome; XLT, X-linked thrombocytopenia; HSCT, hematopoietic stem cell transplantation.

Clinical diagnoses were classified as XLT or WAS based on the correlation between identified *WAS* gene variants, the patient’s medical history, and the clinical scores assigned according to previously established criteria (**Supplementary Table S1**) (10, 19, 21). The most observed clinical manifestations were eczema (76.4%), skin bleeding (e.g., petechiae and ecchymosis, 40.9%), and hematochezia (40.9%), followed by recurrent infections (41.2%). Detailed clinical information for each individual is available in **Supplementary Tables S3A, B**.

**Figure 1:**
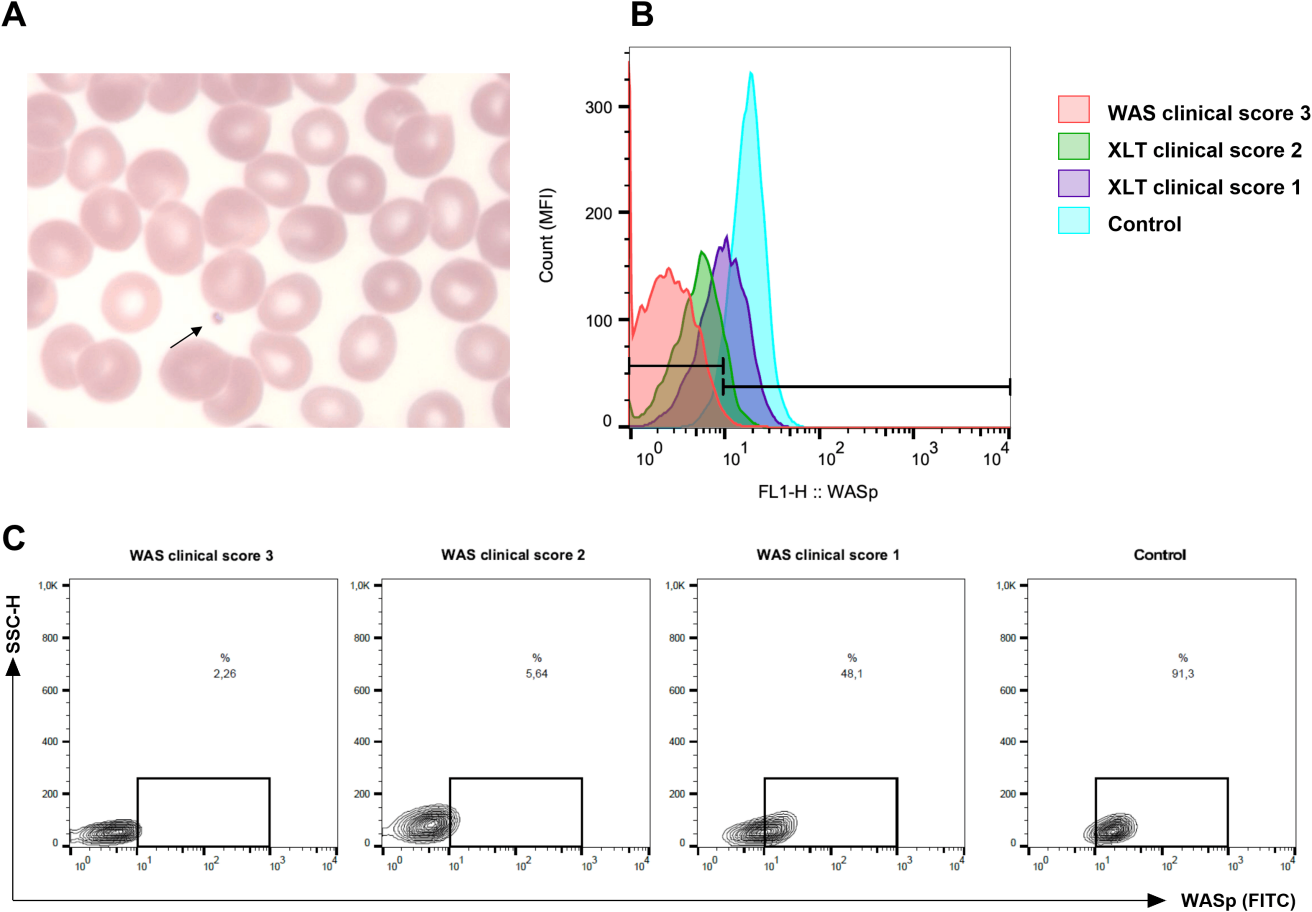
Representation of small platelets and lower expression in Wiskott-Aldrich syndrome protein (WASp) from patients with Wiskott-Aldrich syndrome (WAS) and X-linked thrombocytopenia (XLT). **(A)** Peripheral blood smear from a patient with WAS (P4) showing marked thrombocytopenia and the presence of small platelets (arrow). **(B)** WASp expression analyzed by flow cytometry in lymphocytes. Compared to a normal control (light blue histogram), an XLT patient with a clinical score of 1 (P5, purple), an XLT patient with a clinical score of 2 (P4, green), and a WAS patient with a score of 3 (P1, red) exhibited progressively reduced WASp expression in lymphocytes. The histogram is representative of three independent experiments. A total of six patients were analyzed. **(C)** Representative dot plot corresponding to **(B)**.

Thrombocytopenia was observed in all 31 children enrolled in the study, including those with a confirmed molecular diagnosis of WAS/XLT (22 patients) and those with negative *WAS* gene and *WIPF1* gene sequencing results (9 patients). The presence of small platelets in peripheral blood (**Figure 1A**) was confirmed when samples were available. Among the patients with confirmed WAS/XLT diagnoses, small platelets were observed in 13 out of 14 children (92.8%) for whom this information was available, while one patient, P16A (7.2%), had normal platelet size. Additionally, recurrent infections were reported in 16 out of 22 children (72.7%) with a confirmed WAS/XLT diagnosis (**Supplementary Tables S3A, B**). Detailed clinical manifestations raising suspicion of WAS in the nine patients with negative molecular diagnoses are provided in **Supplementary Table S4**.

WASp expression in lymphocytes was evaluated by flow cytometry whenever samples were available prior to patients undergoing HSCT. Reduced WASp expression was observed in six patients from different families. Notably, patient P16A, the only one without detectable small platelets, exhibited a markedly reduced WASp expression (**Supplementary Tables S3A, B**). **Figures 1B, C** illustrates the difference in WASp expression between three patients with distinct clinical scores and healthy control.

All identified *WAS* gene variants are summarized in **Table 2**. Among the 17 detected variants, seven had been previously reported (24–26), while ten were novel and had not been described in the literature. The most common variation type was frameshift indels (n = 14, 63.6%), followed by missense (n = 5, 22.7%), nonsense (n = 2, 9.1%), and a single splice-site (n = 1, 4.5%). No correlation was observed between *WAS* gene variant type and clinical score (Spearman correlation, r = 0.16).

**Table 2:** Molecular characteristics of WAS patients (Ref Seq: NM_000377.3, NG_007877.1).

Family/patient	Gene location	Nucleotide	Predicted Effect^§^	Variation type	First literature report	Class of WAS gene variant^¶^	Definitive diagnosis	Clinical score	Outcome
F1 P1	exon 1	c.42G>A	p.(Gly3Glu)	*missense*	New variant	I	WAS	3	Wait for HSCT
F2 P2	exon 1	c.140_143del	p.Phe36*	*frameshift*	Jin et al., 2004 (26)	II	WAS	3	HSCT
F3 P3A	exon 1	c.155C>T	p.Arg41*	*nonsense*	Wengler et al., 1995 (25)	II	WAS	5	Death
F3 P3B	exon 1	c.155C>T	p.Arg41*	*nonsense*	Wengler et al., 1995 (28)	II	WAS	5	Death
F4 P4	exon 2	c.168C>T	p.(Thr45Met)	*missense*	Kwan et al., 1995 (24)	I	XLT	2	Follow-up
F5 P5	exon 2	c.207C>T	p.(Pro58Leu)	*missense*	Kwan et al., 1995 (24)	I	XLT	1	Follow-up
F6 P6	exon 2	c.268del	p.(Asn78*48)	*frameshift*	New variant	II	WAS	3	HSCT
F7 P7	exon 3	c.354A>G	p.(Tyr107Cys)	*missense*	Albert et al., 2010 (18)	II	WAS	5^#^	Death
F8 P8	exon 4	c.417_418del	p.(Phe128Cysfs*40)	*frameshift*	New variant	II	WAS	4	HSCT
F9 P9	exon 4	c.431G>A	p.(Glu133Lys)	*missense*	Kwan et al., 1995 (24)	II	WAS	4	HSCT
F10 P10A	exon 7	c.613del	p.(Leu193fs*68)	*frameshift*	New variant	II	WAS	3	HSCT
F10 P10B	exon 7	c.613del	p.(Leu193fs*68)	*frameshift*	New variant	II	WAS	3	HSCT
F11 P11	exon 7	c.743_746del	p.(Asp237fs*21)	*frameshift*	New variant	II	WAS	4	HSCT
F12 P12	intron 8	c.778-2A>G	p.Asp259fs*69	*splice site*	Kwan et al., 1995 (24)	II	WAS	4	HSCT
F13 P13	exon 10	c.1040del	p.Lys336Argfs*108	*frameshift*	New variant	II	WAS	3	HSCT
F14 P14	exon 10	c.1065del	p.(Pro344fs*101)	*frameshift*	New variant	II	WAS	5	Wait for HSCT
F15 P15A	exon 10	c.1187del	p.(Pro385fs*60)	*frameshift*	New variant	II	WAS	3	HSCT
F15 P15B	exon 10	c.1187del	p.(Pro385fs*60)	*frameshift*	New variant	II	WAS	3	HSCT
F16 P16A	exon 10	c.1306_1353del	p.(Leu425Argfs*5)	*frameshift*	New variant	II	WAS	5	Death
F16 P16B	exon 10	c.1306_1353del	p.(Leu425Argfs*5)	*frameshift*	New variant	II	WAS	5	Death
F17 P17A	exon 10	c.1334dup	p.(Leu434Alafs*62)	*frameshift*	New variant	II	WAS	4	HSCT
F17 P17B	exon 10	c.1334dup	p.(Leu434Alafs*62)	*frameshift*	New variant	II	WAS	4	Death

^§^The predicted effects without parenthesis were validated at the cDNA level in this study. ^¶^ *WAS* gene variant classification according to Vallée TC, 2024 (20). Class I includes missense mutations in exons 1 or 2, as well as the c.559 + 5G>A variant. Class II encompasses all other variants of the *WAS* gene. # Patient P7 was initially assigned a score of 3, presenting symptoms at the age of 5 years. At 25 years old, he developed lymphoma and later succumbed to disease-related complications. Following his death, the score was retrospectively adjusted to 5. XLT, X-linked thrombocytopenia; WAS, Wiskott-Aldrich syndrome; HSCT, Hematopoietic stem-cell transplantation; NE, not evaluated.

Regarding previously undescribed *WAS* gene variations, our cohort included one missense change, p.(Gly3Glu) and nine frameshift indels: p.(Asn78fs*48), p.(Phe128Cysfs*40), p.(Leu193fs*68), p.(Asp237fs*21), p.Lys336Argfs*108, p.(Pro344fs*101), p.(Pro385fs*60), p.(Leu425Argfs*5), and p.(Leu434Alafs*62). As cDNA samples were not available for several cases, we utilized the ACMG guideline for clinical interpretation of genetic variants (available at https://wintervar.wglab.org/evds.php) (27), and the frameshift changes were classified as pathogenic, as summarized in **Supplementary Table S5**.

After identifying the patients’ mutations, molecular analyses were performed on 14 mothers to determine whether the mutations were inherited or *de novo*. All screened mothers, except those of Patients 8 and 13, were found to be heterozygous for the alteration. Consequently, Patients 8 and 13 exhibited *de novo* mutations in exons 4 and 10, respectively, both involving deletions that led to premature stop codons.

Regarding patient outcomes, two individuals (9.1%) were awaiting a matched donor for potential HSCT, including one with a novel missense mutation (p.Gly3Glu). Twelve patients (54.5%) underwent HSCT, although one experienced graft failure. Two XLT patients with Class I *WAS* gene variants and mild symptoms have been under follow-up at our center for over 17 years (P4) and 11 years (P5) (Medina et al., 2017). These two patients have decided not to proceed with HSCT thus far, following a shared decision-making process involving pediatricians, hematologists, and the patients and their families. Overall, six fatalities (27.3%) were reported (**Supplementary Tables S3A, B**).

In Family 3, *post-mortem* genetic analysis was performed on two brothers (Patients 3A and 3B), both children of a non-consanguineous couple, who died before the age of two. Both presented with thrombocytopenia, cutaneous and mucosal bleeding, and severe recurrent infections. The initial healthcare providers failed to identify the presence of small platelets. Sequencing of the mother’s DNA revealed a previously described nonsense mutation in the *WAS* gene, p.Arg41* (c.155C>T) (28).

Another fatal case occurred in patient P7, who carried a previously reported missense variant (p.Tyr107Cys) in exon 3 (18), classified as a Class II *WAS* gene variant according to recent classification criteria (20). Initially assigned a clinical score of 3, this patient developed symptoms at the age of five years, including epistaxis, ecchymosis, eczema, and recurrent acute otitis media. He maintained a relatively stable clinical condition until the age of 25 years, when he was diagnosed with lymphoma and, unfortunately, passed away due to complications related to cancer.

**Figure 2:**
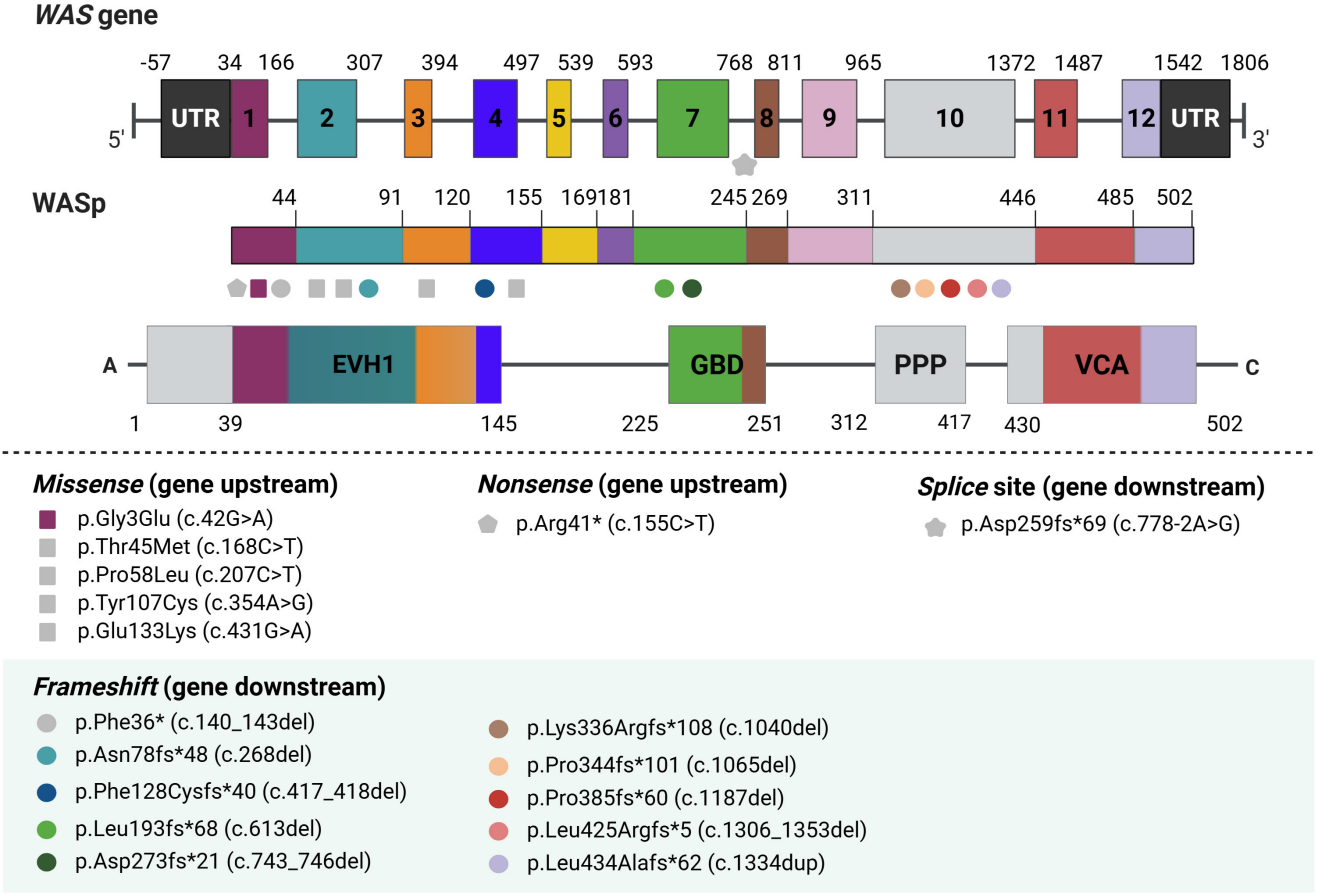
Schematic representation of newly identified WASp mutations. Ten unreported mutations in the WAS gene were identified. Gray symbols indicate previously described WAS variants, while colored symbols represent novel discovered variants. EVH1, Ena/VASP Homology 1; GBD, GTPase-binding domain; PRR, Proline-Rich Region; VCA, Verprolin-homology, Central, and Acidic region.

Family 16 also experienced two fatalities (Patients 16A and 16B). The first child died at three months due to severe bleeding but was not diagnosed at the time. The second child was diagnosed molecularly after presenting with cutaneous-mucosal bleeding and autoimmune manifestations, including hemolytic anemia and immune thrombocytopenic purpura. Subsequently, he developed a cytomegalovirus (CMV) infection and died from related complications. Genetic analysis revealed a novel frameshift mutation in exon 10, p.Leu425Argfs*5, involving the deletion of 46 nucleotides, which impeded the synthesis of the WASp VCA domain (**Figure 2**).

In Family 17, one child died in infancy. The first (Patient 17A) underwent HSCT, while the second (Patient 17B) died at three months following recurrent infections (including sepsis and upper airway infections) and hemorrhages, requiring weekly red blood cell and platelet transfusions. Genetic analysis identified a novel mutation in exon 10, p.Leu434Alafs*62.

## 4 Discussion

This study represents the first comprehensive investigation of WAS-associated molecular changes within the Brazilian population. It highlights the identification of ten novel variants in the *WAS* gene, expanding our understanding of the genetic diversity of this rare disorder. WAS is a severe immunodeficiency disorder with a high risk of mortality. While advances in autologous HSC gene therapy may offer future treatment options, HSCT remains the only curative therapy (17, 29), and the long-term prognosis for untreated patients remains relatively reserved (13, 30).

The clinical-genetic relationship in WAS is complex, with genotype-phenotype correlations not always being straightforward. While thrombocytopenia and small platelet size are classically considered hallmark features of WAS (10, 31), clinical manifestations can differ significantly in severity. In our cohort, small platelets were observed in more than 90% of cases with confirmed molecular diagnosis, consistent with widely accepted diagnostic criteria for WAS (32). However, emerging studies have reported cases of WAS patients with normal platelet volume, highlighting the need for molecular diagnostics to identify atypical presentations (33–35).

Notably, one patient in our cohort (P16A), who presented with severe thrombocytopenia, recurrent infections, and autoimmune manifestations, exhibited normal platelet size despite markedly reduced WASp expression. Genetic analysis revealed a novel frameshift mutation in exon 10 (p.Leu425Argfs*5), reinforcing the complexity of the disease’s presentation. On the other hand, we also observed cases with milder clinical manifestations, typically characterized by a reduced or variable platelet count, categorized as XLT. These patients are often initially misdiagnosed as having ITP (13, 18). This underscores the critical importance of genetic testing in accurately diagnosing WAS/XLT, especially in cases with atypical or milder phenotypes.

While flow cytometry-based assessment of WASp expression in leukocytes offers an useful alternative for screening WAS/XLT (36), we encountered several practical challenges in its application. One major difficulty is the standardization of the technique, which requires careful optimization to ensure consistent and reliable results. Additionally, samples from very young children are often limited in quantity and may be unstable, making it difficult to transport them to centralized laboratories for analysis. A retrospective cohort study on WAS patients demonstrated variability in WASp expression even among individuals with identical mutations, with some cases showing reduced or absent WASp expression despite clinically mild phenotypes (9, 18). In line with these findings, our flow cytometry analysis revealed reduced WASp expression in patients with both severe and mild disease phenotypes (**Figure 1**). These challenges highlight the limitations of flow cytometry as a routine diagnostic tool for WAS, underscoring the need for complementary diagnostic approaches, such as molecular genetic testing, to ensure accurate and timely diagnosis.

The structure of WASp, a key protein involved in actin filament nucleation, is crucial for understanding the pathophysiology of WAS. Mutations in specific regions of the WAS gene, such as those affecting the VCA domain, can severely disrupt the function of WASp, impairing actin dynamics and leading to hallmark immunodeficiency and platelet abnormalities in WAS patients (37, 38). In our study, the identification of nine novel frameshift mutations, many located in exon 10, which encodes part of the proline-rich (PPP) region and the VCA domain (**Figure 2**). These mutations were associated with more severe clinical phenotypes, including early mortality in some patients. For instance, patients from families 16 and 17, both carrying mutations in exon 10, succumbed to their conditions at an early age due to the severity of the mutations (39).

The management of WAS patients remains challenging, particularly when determining the timing of HSCT. Early transplantation is generally recommended, as older age at transplantation is a known risk factor for poorer outcomes (16). However, some studies suggest that the severity of the disease may not always correlate with the WAS score, especially in patients under two years of age (21). In our study, two XLT patients with mild symptoms and a clinical follow-up of over 10 years have not yet undergone HSCT. These decisions were influenced by the patients’ initial presentation, the available clinical data at the time, and the families’ preferences. While current guidelines recommend HSCT before the age of five, our findings highlight the importance of individualized treatment plans based on the patient’s unique clinical course and family considerations. Although post-HSCT complications remain a concern, overall survival has improved significantly over the past decades. Nonetheless, careful, individualized monitoring and timely intervention remain essential for achieving optimal outcomes (40).

Another noteworthy aspect of WAS is its association with an increased incidence of tumors, particularly lymphoma, in affected individuals. Studies have shown that WASp functions as a tumor suppressor in T-cell lymphoma, and its deficiency accelerates lymphoma development (4, 41). Our study reports one patient (P7) who, despite presenting with a mild phenotype, developed lymphoma at a later age, resulting in death due to complications. According to a recent study by Vallée TC et al. (20), which provides updated guidance on WAS variant classification and treatment decisions, our patient, who carries a missense variant in exon 3 (p.Tyr107Cys), a Class II WAS gene variant, should ideally be considered for early HSCT. This emphasizes the critical importance of recent research with larger WAS cohorts and extended follow-up periods, which could help refine treatment strategies. Studies such as that by Albert et al. (16) bring attention to the need for early HSCT consideration, even for patients with lower clinical scores or milder phenotypes. Indeed, the growing body of evidence from recent studies calls for refining HSCT strategies and making individualized treatment decisions based on the unique clinical course of each patient.

We acknowledge the limitations of our study. Given the rarity of the disease and the limited sample size, the broader applicability of our results may be constrained. Additionally, the fact that patients were referred from multiple centers introduces potential challenges, such as missing data, and combined with the patients’ age and disease severity, this restricted the availability of biological samples prior to HSCT. Furthermore, longitudinal follow-up was not always manageable. Despite these limitations, our study provides valuable insights into the genetic diversity and clinical spectrum of WAS in the Brazilian population, contributing to a better understanding of the disease.

In conclusion, this study provides the first comprehensive exploration of the genetic and clinical features of WAS in the Brazilian population, significantly expanding our understanding of this rare and complex disorder. By identifying ten novel mutations in the WAS gene, we contribute to the growing body of knowledge regarding the genetic diversity and phenotypic spectrum of WAS, highlighting the importance of early molecular diagnosis, particularly for atypical or milder presentations. Our findings emphasize that WAS is a multifaceted disease with clinical manifestations that do not always correlate directly with the genotype, making accurate diagnosis and timely treatment crucial.

## Data Availability

The datasets presented in this study can be found in online repositories. The names of the repository/repositories and accession number(s) can be found below: https://portal.fcm.unicamp.br/biorrepositorio-laboratorio/, LIDH’s Laboratory Biorrepository.
