# Supplementary material for: New insights into Wiskott-Aldrich syndrome: ten novel WAS mutations and their clinical impact in a Brazilian cohort
Source: Front Immunol. 2025 Jul 31;16:1585594. doi: 10.3389/fimmu.2025.1585594 (PMC12350080; doi:10.3389/fimmu.2025.1585594)
Supplement: Supplementary file 1 [file DataSheet1.pdf]

Supplementary Material

1 Supplementary Data

Supplementary Table S1. Clinical score to classify Wiskott-Aldrich Syndrome according to Zhu Q, et al. (1995) and Ochs HD (2009), with additional criteria proposed by Mahlaoui N, et al. (2013).

**Disease phenotype**	XLT		Classic WAS phenotype		Severe WAS phenotype	Early-onset severe WAS
**Severity score**	1	2	3	4	5	EOS
Onset before the age of 2 y	-/+	-/+	-/+	-/+	-	+
Thrombocytopenia	+	+	+	+	+	+
Microthrombocytes	+	+	+	+	+	+
Eczema	-	-/+	+	+/++	+ / ++	+/++
Immunodeficiency	-	-/+	+	+	+	+
Infections	-	-/+	+	+ / ++	+ / ++	+
Autoimmunity and/or vasculitis and/or neoplasia	-	-	-	-	+	+/++
Severe refractory thrombocytopenia (SRT)*	-	-	-	-	+	+**

*SRT is defined as a platelet count that never increased above 10 G/L (even 1 hour after platelet transfusion) and bleeding (regardless of the patient's antiplatelet antibody status). **Not observed in all patients. Score: -, absent; -/+, moderate; +, present; ++, present and severe; XLT, X-linked thrombocytopenia; WAS, Wiskott-Aldrich Syndrome; EOS, early-onset severe WAS phenotype.

Supplementary Table S2. Oligonucleotides designed for amplification of *WAS* gene and *WIPF1* gene.

Exon	Forward primer (5' - 3')	Reverse primer (5' – 3')	Annealing Temp (ºC)
***WAS* gene gDNA NCBI Reference Sequence: NG_007877.1**			
**Exon 1 and 2**	TCCTGTTCCCTTGCTGCTCAT	GGTTTGCGGGTTGAGAACTG	60
**Exon 3 to 6**	GAGGCTCCCAAATCCAGACAC	GAGACACAGGGAAGAATCAGCA	62
**Exon 7**	GTTGGTTGGTAAGTGGGTCA	CTTGTTCATGTGCCCCTCTG	60
**Exon 8 and 9**	AAGGAAGGGCAGTGAGGATT	CCACTGACCAACTCCTGACTG	59
**Exon 10**	TCAGTCAGGAGTTGGTCAGTGG	TCTCTCCCACACACTCCAGTC	57
**Exon 11**	CAGGCCTTAGGGATTCAGTG	TGGTGGTGACTGCTGGGATTG	60
**Exon 12**	GTCCAGTCCTCACCTCCCAG	TTGAAAGACAGAGGGGCAG	59
***WAS* gene cDNA NCBI Reference Sequence: NM_000377.3**			
**Fragment 1**	TGCACCCAGAGCCTCGCCAG	CCGCTTGGCAGTCATCTCCAGC	60
**Fragment 2**	CCTTCGCTGGAGATGACTGCC	TCACGTCAAATCCATTCTGGGG	60
**Fragment 3**	CCCCAGAATGGATTTGACGTGA	GGGGTCTTGTTCAGCTGAATTC	55
**Fragment 4**	GGAATTCAGCTGAACAAGACCC	AGGGCCTTGGGATTGTTGGGTG	60
***WIPF* gDNA NCBI Reference Sequence: NG_032009.1**			
**Exon 1**	GCCTCTGACTCAGTATGTCA	CTTCCCTGTAGCTGATAAATG	55
**Exon 2**	GTGGGAAAGTGAAGACAGATT	TTTTGGCTTCATTTGGCTTGC	55
**Exon 3**	CCCAAGAGATGACTACTTGTT	ATAAACTGTTCCTCTCCATGG	55
**Exon 4**	TATGGTTTACCTGGGCCAGA	TTGTCATCCAAGGCCCTGC	55
**Exon 5-I**	TCAGGGTTCCTGATTTCCTAA	TTGTCATCCAAGGCCCTGC	55
**Exon 5-II**	TTTGGGAGGAGGCTCAATAC	GCTGGGTTTTGGAAGCAACT	55
**Exon 6**	GGATGAAGGTTCTGCACTAA	TTTACCATTTAGTTGCTGCCC	60
**Exon 7**	CACATTGTCTGTCACTGAAGA	CTATATAGCCCGAGTGTCAC	55
**Exon 8**	CCACGATGAGAACATCAGCT	CACACACGCATATTCCCACT	64
***WIPF1* gene cDNA NCBI Reference Sequence: NM_001077269.1**			
**Fragment 1**	TCCCTGCAGTGTGGCTTCTTAG	ATCATTATCCCTGTTGGCCGTG	62
**Fragment 2**	TACTGGACAAACCTAAAGGAGC	GAGGCTTGTTGTTCTGAGGAGG	60
**Fragment 3**	CTAGTACTCCAAGACCCATTC	TCTGCTTTCCCACTCATCTTC 56	56
**Fragment 4**	GGCCCCCTCCCACCACCTCC	CAAGGGAAGAAGCAGGGAGGAG	65

frontiers

Supplementary Table S3A. Clinical characteristics of the patients diagnosed with Wiskott-Aldrich syndrome.

Characteristics	P1	P2	P3A	P3B	P4*	P5*	P6	P7	P8	P9	P10A	P10B
**Mother in heterozygosis**	+	+	+	+	+	+	NE	NE	-	+	+	+
**Age at first visit (month)**	9	1	1	-	9	1	1	60	17	1	2	1
**Platelet count ($x10^9/l$)**	56	35-58	6	60	27-120	12-64	34-56	6	11	7-23	20	<20
**Mean Platelet Volume (fl)**	7.9	5.3-6.7	NE	NE	5.6-6.5	6-6.2	4.3-6.1	NE	NE	7.4-12.9	NE	NE
**Small Platelets**	+	+	NE	NE	+	+	+	NE	NE	+	+	NE
**WASp lymphocytes expression§**	NE	A	NE	NE	R	R	NE	NE	NE	NE	NE	NE
**Cutaneous-mucous bleeding**	+	-	-	-	+	-	-	+	+	-	-	-
**Petechiae/Bruise**	+	-	-	+	-	+	-	+	+	+	+	+
**Hematochezia**	+	+	-	+	-	+**	-	-	+	+	-	+
**Eczema**	+	+	+	+	-	-	+	+	+	-	+	+
**Epistaxis**	+	-	+	+	-	-	-	+	+	-	-	-
**Skin infections**	-	-	-	+	-	-	-	-	+	-	-	-
**Allergies**	-	-	-	-	-	-	-	-	-	-	-	-
**Pneumonia**	-	-	+¶	-	-	-	-	-	-	+	-	-
**Recurrent Infections**	-	-	-	-	-	-	-	+	+	-	+	-
**Respiratory Infection**	-	-	-	-	-	-	-	-	-	-	+	-
**Severe neutropenia**	-	-	-	-	-	-	-	-	-	-	-	-
**Rhinitis/Sinusitis**	-	-	-	-	-	-	-	-	-	-	-	-
**Otitis**	-	+	-	-	-	-	-	+	-	+	-	-
**Hematemesis**	-	-	-	-	-	-	-	-	-	-	-	-
**Abscesses**	-	-	-	-	-	-	-	-	+	-	-	-
**Autoimmune manifestations**	-	-	-	-	-	-	-	-	+	-	-	-
**Initially diagnosed as ITP**	Yes	No	No	No	Yes	Yes	No	No	No	Yes	No	No

*X-linked thrombocytopenia; **accidental finding; ***gangrenous pyoderma; §WASp lymphocytes expression by flow cytometry. ¶sepsis; NE, not evaluated; WASp, Wiskott-Aldrich protein; R, reduced; A, absent; ITP, immune thrombocytopenia.

Supplementary Table S3B. Clinical characteristics of the patients diagnosed with Wiskott-Aldrich syndrome.

Characteristics	P11	P12	P13	P14	P15A	P15B	P16A	P16B	P17A	P17B
**Mother in heterozygosis**	+	+	-	+	NE	NE	+	+	+	+
**Age at first visit (month)**	7	2	11	14	12	18	3	3	1	5
**Platelet count ($x10^9$/l)**	15	15-68	7-33	18	26	24	2-16	<10	5	<10
**Mean Platelet Volume (fl)**	NE	6-7.6	5.1-6.5	6.8	NE	NE	9.2-11.9	NE	7.5	NE
**Small Platelets**	NE	+	+	+	+	+	-	NE	+	NE
**WASp lymphocytes expression[§]**	NE	R	A	NE	NE	NE	R	NE	NE	NE
**Cutaneous-mucous bleeding**	-	-	-	+	-	-	+	-	-	-
**Petechiae/Bruise**	-	-	-	+	+	+	+	+	-	+
**Hematochezia**	+	-	-	+	-	-	-	+	+	+
**Eczema**	+	+	-	+	+	+	-	-	+	-
**Epistaxis**	-	-	+	+	+	+	-	+	-	-
**Skin infections**	+[***]	+	+	+	-	-	-	-	-	+
**Allergies**	-	+	-	+	-	-	+	-	+	-
**Pneumonia**	-	-	-	-	-	-	-	-	-	+
**Recurrent Infections**	-	-	-	+	+	+	+	-	+	+
**Respiratory Infection**	+	-	-	+	-	+	-	-	+	-
**Severe neutropenia**	-	-	+	-	-	-	+	-	-	-
**Rhinitis/Sinusitis**	-	-	+	-	-	-	-	-	-	-
**Otitis**	-	+	-	-	-	-	-	-	-	-
**Hematemesis**	-	-	+		-	-	-	-	-	-
**Abscesses**	+	+	-	-	-	-	-	-	-	-
**Autoimmune manifestations**	-	+	+	+	-	-	+	-	-	-
**Initially diagnosed as ITP**	No	No	No	No	No	No	No	No	No	No

[*]X-linked thrombocytopenia; [**]accidental finding; [***]gangrenous pyoderma; [§]WASp lymphocytes expression by flow cytometry. [¶]sepsis; NE, not evaluated; WASp, Wiskott-Aldrich protein; R, reduced; A, absent; ITP, immune thrombocytopenia.

Supplementary Table S4. Clinical characteristics of cases suspected of Wiskott-Aldrich syndrome but with negative molecular diagnoses.

Characteristics	F18 P18	F19 P19	F20 P20	F21 P21	F22 P22	F23 P23	F24 P24	F25 P25	F26 P26
**Age at first visit (years)**	8	2 months	7	6	10	7	1	6	12
**Platelet count (x10^9/l)**	30-80	21	6	24	40	46-125	109	8	30-80
**Mean Platelet Volume (fl)**	11.4	NE	6.7	9.1	6-9	6.5	6.7	7.9	11
**Small Platelets**	+	+	+	-	-	+	+	+	+
**Cutaneous-mucous bleeding**	-	-	-	-	-	-	-	+	-
**Petechiae/Bruise**	-	-	+	+	-	+	-	+	+
**Hematochezia**	-	-	+	-	-	-	-	-	-
**Eczema**	+	+	-	-	-	-	-	+	-
**Epistaxis**	-	-	-	+	-	+	+	-	-
**Skin infections**	+	-	-	-	-	+	-	-	-
**Allergies**	-	-	-	-	-	+	+	-	-
**Pneumonia**	-	-	-	+	+	-	-	-	-
**Recurrent Infections**	-	-	-	-	-	+	+	+	+
**Respiratory Infection**	-	-	-	+	-	-	+	-	-
**Severe neutropenia**	-	-	-	+	+	-	-	-	+
**Rhinitis/Sinusitis**	-	-	-	-	-	-	-	-	-
**Otitis**	-	-	+	-	-	+	-	-	-
**Hematemesis**	-	-	-	-	-	-	-	-	-
**Abscesses**	-	-	-	-	-	-	-	-	-

NE, not evaluated.

Supplementary Table S5. Classification of genetic variants observed according to the American College of Medical Genetics (ACMG).

Family/Patient	Predicted Effect[§]	Variation type	ACMG classification[†]	AMCG criteria	First literature report
F1 P1	p.(Gly3Glu)	missense	Uncertain Significance	PM2, PP2, BP4, PP4	New variant
F2 P2	p.Phe36*	frameshift	Pathogenic	PVS1, PM2, PM4	Jin *et al*., 2004
F3 P3A and P3B	p.Arg41*	nonsense	Pathogenic	PVS1, PM1, PM4, PP1, PP3, PP4	Wengler *et al*., 1995
F4 P4	p.(Thr45Met)	missense	Likely benign	PM2, BP4, BP7	Kwan *et al*., 1995
F5 P5	p.(Pro58Leu)	missense	Likely benign	PM2, BP4, BP7	Kwan *et al*., 1995
F6 P6	p.(Asn78fs*48)	frameshift	Pathogenic	PVS1, PM2, PM4, PP4	New variant
F7 P7	p.(Tyr107Cys)	missense	Likely benign	PM2, PBP4, BP7	Albert *et al*., 2010
F8 P8	p.(Phe128Cysfs*40)	frameshift	Pathogenic	PVS1, PM2, PM4, PP4	New variant
F9 P9	p.(Glu133Lys)	missense	Uncertain Significance	PM2, PP2, PP4, PP5	Kwan *et al*., 1995
F10 P10A and P10B	p.(Leu193fs*68)	frameshift	Pathogenic	PVS1, PM1, PM4, PP4	New variant
F11 P11	p.(Asp237fs*21)	frameshift	Pathogenic	PVS1, PM4, PP4	New variant
F12 P12	p.Asp259fs*69	splice site	Pathogenic	PVS1, PM4, PP4	Kwan *et al*., 1995
F13 P13	p.Lys336Argfs*108	frameshift	Pathogenic	PVS1, PS2, PM4, PP4	New variant
F14 P14	p.(Pro344fs*101)	frameshift	Pathogenic	PVS1, PM4, PP4	New variant
F15 P15A and P15B	p.(Pro385fs*60)	frameshift	Pathogenic	PVS1, PM4, PP4	New variant
F16 P16A and P16B	p.(Leu425Argfs*5)	frameshift	Pathogenic	PVS1, PM4, PP4	New variant
F17 P17A and P17B	p.(Leu434Alafs*62)	frameshift	Pathogenic	PVS1, PM4, PP4	New variant

[§] The predicted effects without parenthesis were validated at the cDNA level in this study. [†]Genetic interpretation was established following AMCG-AMP 2015 guideline criteria (Li et al., 2017).

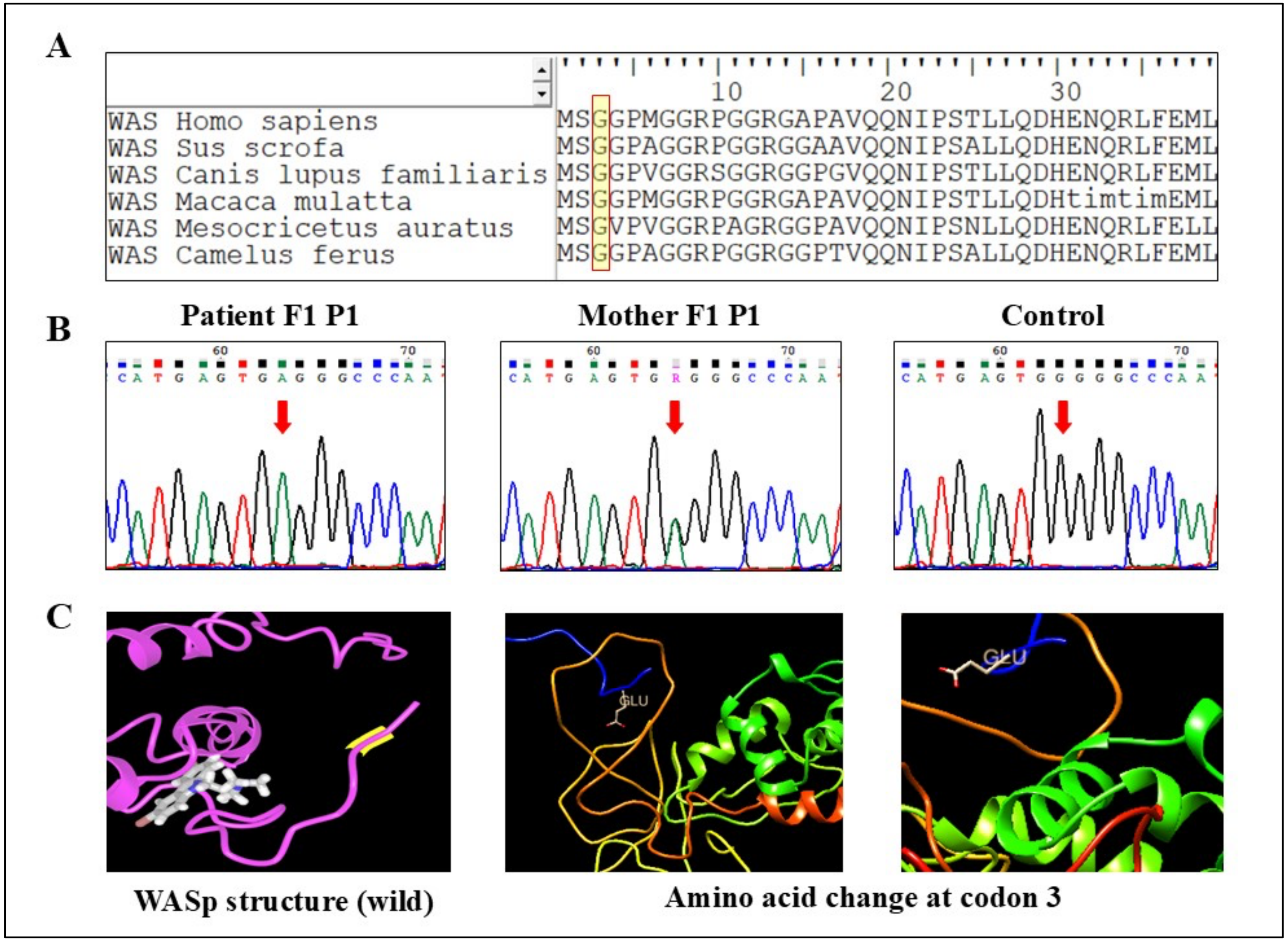

Supplementary Figure 1. In silico analysis of the novel missense variant p.Gly3Glu encoded by the *WAS* gene (NM_000377.3). (A) For the alignment of vertebrate mammalian protein sequences, we used the BioEdit tool (Hall, 1999) and applied a global alignment approach. The sequences were retrieved from the NCBI Orthologs database. (B) Electropherogram showing the missense mutation p.Gly3Glu in the *WAS* gene. The altered nucleotide is highlighted, confirming the presence of the variant at the genomic level. (C) The homology modeling of the mutated WAS protein (NM_000377.3) was performed using the Modeller 10.4 software (Webb et al, 2016), based on the crystal structure PDB: 1EJ5, available in the Protein Data Bank (PDB). Multiple 3D structural models were generated, and the final model (WAS.B99990002.pdb) was selected based on the lowest DOPE score (-20054.23). The resulting structures were validated and visualized using UCSF Chimera (Pettersen et al, 2004), allowing for a detailed analysis of the molecular conformation and overall model quality.

References included in the Supplementary material

Adzhubei IA, Schmidt S, Peshkin L, Ramensky VE, Gerasimova A, Bork P, Kondrashov AS, and Sunyaev SR. A method and server for predicting damaging missense mutations. *Nat Methods* (2010) 7:248. doi:10.1038/nmeth0410-248

Hall TA. BioEdit: A User-Friendly Biological Sequence Alignment Editor and Analysis Program for Windows 95/98/NT. *Nucleic Acids Symp Ser* (1999) 41:95–98

Li Q and Wang K. InterVar: Clinical Interpretation of Genetic Variants by the 2015 ACMG-AMP Guidelines. *Am J Hum Genet* (2017) 100:267–280. doi:10.1016/j.ajhg.2017.01.004

Mahlaoui N, Pellier I, Mignot C, Jais J-P, Bilhou-Nabéra C, Moshous D, Neven B, Picard C, de Saint-Basile G, Cavazzana-Calvo M, et al. Characteristics and outcome of early-onset, severe forms of Wiskott-Aldrich syndrome. *Blood* (2013) 121:1510–1516. doi:10.1182/blood-2012-08-448118

Ochs HD. Mutations of the Wiskott-Aldrich Syndrome Protein affect protein expression and dictate the clinical phenotypes. *Immunol Res* (2009) 44:84–88. doi:10.1007/s12026-008-8084-3

Pettersen EF, Goddard TD, Huang CC, Couch GS, Greenblatt DM, Meng EC, and Ferrin TE. UCSF Chimera—a visualization system for exploratory research and analysis. *J Comput Chem* (2004) 25:1605–1612. doi:10.1002/jcc.20084

Webb B and Sali A. Comparative protein structure modeling using MODELLER. *Curr Protoc Bioinformatics* (2016) 54:5.6.1–5.6.37. doi:10.1002/cpbi.3

Zhu Q, Zhang M, Blaese RM, Derry JM, Junker A, Francke U, Chen SH, and Ochs HD. The Wiskott-Aldrich syndrome and X-linked congenital thrombocytopenia are caused by mutations of the same gene. *Blood* (1995) 86:3797–3804
